# Epidemiology of maxillofacial injuries during monsoon and non-monsoon season in India: a data-based retrospective study from a tertiary care dental teaching hospital

**DOI:** 10.12688/f1000research.134532.1

**Published:** 2023-10-19

**Authors:** Anand Deep Shukla, Srikanth G, Kalyan Chakravarthy, Ayusha Kaushal, Hariti M Udeshi

**Affiliations:** 1 Department of Oral and Maxillofacial Surgery, Manipal College of Dental Sciences, Manipal , Manipal Academy of Higher Education, Manipal, Karnataka, 576104, India; 2 Department of Public Health Dentistry, Manipal College of Dental Sciences, Manipal, Manipal Academy of Higher Education, Manipal, Karnataka, 576104, India

**Keywords:** Maxillofacial injuries, Road Traffic Accidents

## Abstract

**Background:** Maxillofacial Injury (MFI) is a major public health concern that is multifactorial in etiology-road traffic accidents (RTAs), falls and violence. RTAs are the major cause of maxillofacial injuries (MFIs) in countries like India. Recent studies have shown that maxillofacial fractures (MFF) constitute a significant proportion of facial injuries seen in hospitals (56.5%). The incidence of maxillofacial fractures can vary depending on several factors, including age, gender, and environmental factors. Of particular concern is the impact of seasonal variations, such as the monsoon season, which lead to high incidence of maxillofacial fractures due to hazardous conditions.

**Methods:** A retrospective review of medical records was done in a tertiary-care dental teaching hospital was done.

**Results:** Data of 200 subjects including 154 males (77%) and 46 females (23%) with a mean age of 35.38 ± 16.541 years; age range: 1 – 80 years was analyzed. A total of 200 MFI’s were recorded between 2021 and 2022. Soft tissue injuries were reported in 37.5% of the cases in non-monsoon season and 42.3% of the cases during the monsoon season. Dentoalveolar fractures were reported in 6.2% of the cases during the non-monsoon seasons and 7.7% during the monsoon season. In this study, mandible was the most fractured bone (n=104,52%) followed by zygomatic complex (n=50, 25%). The frequently observed pattern among mandibular fracture was condyle 8.3% during the non-monsoon season and 2.9% during the monsoon season).

**Conclusions**: The results of the study indicate that mandibular fractures are most commonly seen in maxillofacial fractures, followed by fractures of the zygomatic complex. The study also reveals a higher incidence of soft tissue injuries and dentoalveolar fractures during the monsoon season. Further research is warranted to explore the factors that contribute to the seasonal variation in maxillofacial fractures for effective interventions to reduce their occurrence.

## Introduction

Maxillofacial injury (MFI) is a broad term used to describe any form of damage or trauma to the structures of the face, including the jaw, teeth, and facial bones and associated soft tissues. ^1^ Maxillofacial fractures (MFF), on the other hand, specifically refer to fractures or breaks in one or more of the facial bones, such as the mandible or zygomatic complex. It is important to note that while MFFs are a type of MFI, not all MFIs involve fractures.

MFIs are a significant public health concern, with a wide spectrum of severity that can range from minor injuries to life-threatening conditions. ^2^ The incidence of maxillofacial fractures can vary depending on several factors, including age, gender, and environmental factors.

Recent studies have demonstrated that maxillofacial fractures are among the most frequent types of facial injuries seen in hospitals 56.5%. ^3^^,^^4^ These fractures can involve different components of the facial skeleton, including the mandible, maxilla, zygoma, and orbital bones. ^3^^,^^4^

Additionally, it is important to consider how environmental factors may affect the number of maxillofacial fractures in hospital settings. The monsoon season, for example, may result in an increase in maxillofacial fractures owing to slippery roads and pavements, reduced visibility, and fallen trees and electrical wires.

Healthcare providers must be aware of these trends and take steps to predict and manage maxillofacial injuries. Therefore, this research paper aims to compare the incidence and pattern of maxillofacial trauma in a hospital-based setting with a focus on environmental factors.

## Methods

### Study design

The present study was conducted in the Department of Oral and Maxillofacial Surgery, Manipal College of Dental Sciences Manipal, Udupi, Karnataka, India. This study was based on a systematic computer-assisted database search that allowed extraction of retrospective data of the patients who reported to our outpatient unit with injuries from RTA from December 2021 to December 2022.

Data collection was initiated only after requisite approvals were obtained from the Scientific Committee and the Institutional Ethics Committee of Hospital (IEC:57/2022).

The requirement for informed consent was waived in view of the retrospective nature of the study and there being no direct contact with the study subjects. This study did not involve any intervention or therapy, and the research involved no risks to the subjects. Subjects’ names and identity were not disclosed in any way during or after this database review study. Subjects were identified by subject ID numbers only, and hence, patient data confidentiality has been maintained.

### Subject screening, inclusion and exclusion criteria

Patients of both sexes and all age groups with clinically and radiographically diagnosed maxillofacial fractures (with or without contiguous bodily fractures/injuries) were included in this study. Patients with incomplete records were excluded from the study. Patients with isolated skull fractures and only minor superficial soft tissue injuries were excluded from our study.

No formal sample size was calculated and all 200 patients who met the inclusion criteria during the study period were included.

### Data collection

The hospital records were assessed and data on age, sex and season during which injury occurred was collected. Data was also recorded for anatomic location of facial fractures, associated soft tissue and dentoalveolar injury. In records fractures had been classified as fractures of the mandible, zygomatic-maxillary complex (OZMC), orbital floor, nose, Lefort I,II,III and naso-orbital-ethmoid (NOE) fracture. Mandibular fractures included fractures of the symphysis, Para symphysis, body, angle, ramus, coronoid, and condyle ( Tables 3 and 4).

### Statistical analysis

Statistical analysis was performed using SPSS-20.0 for the Windows Statistical Package (IBM Corporation, Armonk, NY, USA) and a p value of ≤0.05 was considered statistically significant.

Descriptive data were presented as mean ± SD or number (%), unless specified. Univariate analysis was done by Student’s *t*-test. Data for incidence of soft tissue injuries, dentoalveolar Injuries and various facial fractures were compared between the two seasons i.e., monsoon and non-monsoon.

## Results

**Figure 1.**
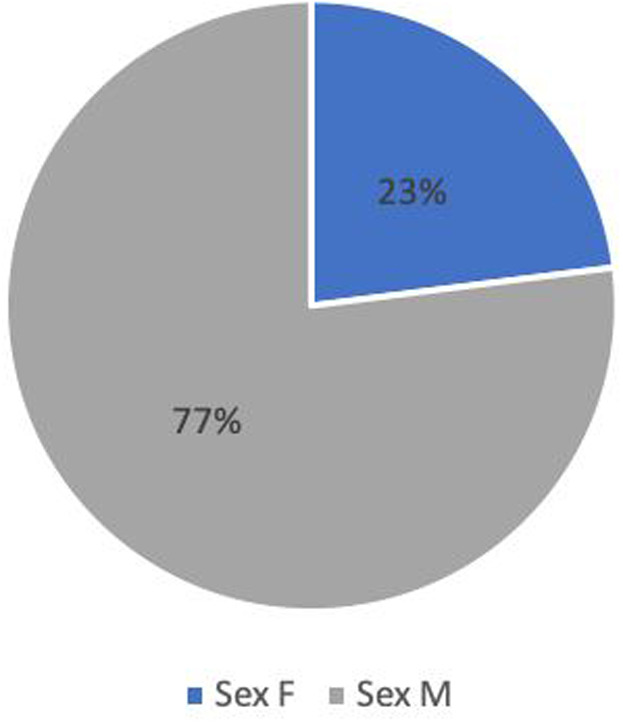
Pie chart showing the sex distribution of the studied maxillofacial trauma cases.

In one year, period between December 2021 to December 2022, data of a total of 200 subjects were recorded and analyzed. Out of these, 154 (77%) were males while only 47 (23%) were females ( Table 2). Male to female ratio was 3.28:1 (rounded to two decimal places) ( Figure 1). The mean age of study population was 35.38 ± 16.541 years (age range: 1-80 years) ( Table 1).

**Table 1.** Mean age of distribution.

	N	Minimum	Maximum	Mean	Std. Deviation
Age	200	1	80	35.38	16.541

**Table 2.** Sex distribution.

		Frequency	Percent
Sex	F	46	23.0
	M	154	77.0

**Table 3.** Distribution of maxillofacial fractures in Monsoon and Non-monsoon seasons.

	Non-monsoon season		Monsoon season		P-value
	N	%	N	%	
Soft Tissue Injury					
Absent	60	62.5%	60	57.7%	0.488
Present	36	37.5%	44	42.3%	
Dentoalveolar Injury					
Absent	90	93.8%	96	92.3%	0.69
Present	6	6.2%	8	7.7%	

**Table 4.** Distribution of various types of fractures in Monsoon and Non-monsoon season.

		Monsoon				P-value
		0		1		
		N	%	N	%	
SOFT TISSUE INJURY	0	60	62.5%	60	57.7%	0.488
	1	36	37.5%	44	42.3%	
DENTOALVEOLAR	0	90	93.8%	96	92.3%	0.69
	1	6	6.2%	8	7.7%	
LEFORT I	0	95	99.0%	100	96.2%	0.371
	1	1	1.0%	4	3.8%	
LEFORT II	0	96	100.0%	103	99.0%	>0.99
	1	0	0.0%	1	1.0%	
LEFORT III	0	92	95.8%	99	95.2%	>0.99
	1	4	4.2%	5	4.8%	
FRONTAL BONE	0	87	90.6%	101	97.1%	0.053; Sig
	1	9	9.4%	3	2.9%	
PALATAL BONE	0	93	96.9%	103	99.0%	0.352
	1	3	3.1%	1	1.0%	
TEMPORAL BONE	0	92	95.8%	103	99.0%	0.147
	1	4	4.2%	1	1.0%	
ZYGOMATIC ARCH	0	72	75.0%	98	94.2%	<0.001; Sig
	1	24	25.0%	6	5.8%	
NOE	0	95	99.0%	102	98.1%	>0.99
	1	1	1.0%	2	1.9%	
FZ	0	93	96.9%	104	100.0%	0.109
	1	3	3.1%	0	0.0%	
PYRIFORM	0	94	97.9%	102	98.1%	>0.99
	1	2	2.1%	2	1.9%	
ZMC	0	61	63.5%	87	83.7%	0.001; Sig
	1	35	36.5%	17	16.3%	
ORBITAL RIM	0	89	92.7%	104	100.0%	0.005; Sig
	1	7	7.3%	0	0.0%	
ORBITAL FLOOR	0	73	76.0%	92	88.5%	0.021
	1	23	24.0%	12	11.5%	
ORBITAL WALL	0	94	97.9%	103	99.0%	0.609
	1	2	2.1%	1	1.0%	
SPHENOID BONE	0	96	100.0%	103	99.0%	>0.99
	1	0	0.0%	1	1.0%	
TYMPANIC PLATE	0	95	99.0%	103	99.0%	>0.99
	1	1	1.0%	1	1.0%	
NASAL BONE	0	85	88.5%	98	94.2%	0.149
	1	11	11.5%	6	5.8%	
SINUS WALL	0	93	96.9%	104	100.0%	0.109
	1	3	3.1%	0	0.0%	
PTERYGOID PLATE	0	95	99.0%	104	100.0%	0.48
	1	1	1.0%	0	0.0%	
SYMPHYSIS	0	96	100.0%	101	97.1%	0.247
	1	0	0.0%	3	2.9%	
PARASYMPHYSIS	0	94	97.9%	100	96.2%	0.684
	1	2	2.1%	4	3.8%	
ANGLE	0	94	97.9%	101	97.1%	>0.99
	1	2	2.1%	3	2.9%	
BODY	0	91	94.8%	99	95.2%	>0.99
	1	5	5.2%	5	4.8%	
RAMUS	0	95	99.0%	103	99.0%	>0.99
	1	1	1.0%	1	1.0%	
CONDYLE	0	88	91.7%	101	97.1%	0.091
	1	8	8.3%	3	2.9%	
CORONOID	0	94	97.9%	104	100.0%	0.139
	1	2	2.1%	0	0.0%	

0: Indicates Fracture absent; 1: Indicates fracture present.

## Discussion

Maxillofacial trauma can be caused by various factors including falls, assaults, sporting injuries and road traffic accidents. Of these, road traffic accidents and monsoon weather conditions have been identified as significant contributors to the incidence of maxillofacial injuries. Given the increasing prevalence of road traffic accidents and unpredictable weather patterns, it is important to understand the epidemiology of maxillofacial injuries and identify any underlying patterns or trends that may exist to help reduce their incidence.

Some regions show a correlation between monsoon season and an increase in facial fractures, possibly due to broken and slippery roads/pavements, ^5^^,^^6^ falling objects, electrical wires, poor visibility and increased traffic congestion. This can lead to an increase in road traffic accidents and facial injuries. However, there is evidence to suggest that monsoon periods may have fewer cases of facial fractures as people may prefer to stay indoors in anticipation of rain. ^7^

Understanding these trends and patterns can help develop preventive measures to improve public safety, especially in areas where monsoons are severe.

The incidence of maxillofacial fractures is higher in developing countries than in developed ones due to several factors such as the lack of adequate safety measures, poor road infrastructure and social factors. ^5^^,^^8^

The increase in both the frequency and severity of maxillofacial injuries can be linked to the high dependence on road transportation and the growth of socio-economic activities in developing countries. Contributing to these injuries are poor road safety awareness, unsuitable road conditions, underdeveloped motorways, speeding, outdated vehicles without safety features, and a lack of helmets and seat belts, as well as violations of traffic laws. ^5^^,^^9^ All these factors come together to create a high number of road traffic accidents in developing countries.

Men show higher incidence for MFI’s due to their greater involvement in high-risk activities, outdoor activities, anatomical differences, and behavioral differences such as risk-taking in comparison to women as only few of them drive a vehicle. This has resulted in an increase in the male: female ratio this is found to be consistent with the findings reported in other research papers. ^4^^,^^5^^,^^8^

On analyzing the data, we found that soft tissue injury and dentoalveolar injury are not significantly associated with monsoon season, while zygomatic arch, zygomaticomaxillary complex, frontal, orbital floor and orbital rim fractures are significantly more common during monsoon season.

In this study the incidence of maxillofacial trauma due to RTA during the monsoon season was found to be lesser but the severity of trauma was found to be greater. ^7^

Our study was conducted in an environment with better road infrastructure and easy access to immediate medical care, which may have contributed to a lower incidence of maxillofacial trauma. This contrasts with previous studies that have reported a higher incidence of maxillofacial trauma in low-income countries with poor infrastructure and limited access to healthcare services.

Future studies that are planned shall have a database which includes patients over a longer period of time so that the database is much wider.

## Data Availability

Figshare: Data for the study on epidemiology of maxillofacial trauma, https://doi.org/10.6084/m9.figshare.23558628.v2. ^10^
- Data updated.xlsx - data legend.docx Data are available under the terms of the Creative Commons Attribution 4.0 International license (CC-BY 4.0).
