# Supplementary material for: Epidemiology of maxillofacial injuries during monsoon and non-monsoon season in India: a data-based retrospective study from a tertiary care dental teaching hospital
Source: F1000Res. 2024 Mar 4;12:1377. Originally published 2023 Oct 19. [Version 2] doi: 10.12688/f1000research.134532.2 (PMC10997983; doi:10.12688/f1000research.134532.2)
Supplement: Peer review history

# Reviewer report: Reviewer response for version 2

**DOI:** 10.5256/f1000research.163022.r252544

**Published:** 2024-04-05

**Authors:** Rui Santos

**Affiliations:** 1 School of Technology and Management, Polytechnic Institute of Leiria, Leiria, Portugal; 2 Integrated Researcher at the Statistics and Applications Center of the University of Lisbon, Lisboa, Portugal

The authors considered the majority of my comments during the manuscript review, leading to enhancements in its quality.

Is the work clearly and accurately presented and does it cite the current literature?

Yes

If applicable, is the statistical analysis and its interpretation appropriate?

Partly

Are all the source data underlying the results available to ensure full reproducibility?

Partly

Is the study design appropriate and is the work technically sound?

Partly

Are the conclusions drawn adequately supported by the results?

Partly

Are sufficient details of methods and analysis provided to allow replication by others?

Partly

Reviewer Expertise:

Statistical inference, data science, meta-analysis and epidemiology, mainly applied to the areas of Medicine and Forensic Science.

I confirm that I have read this submission and believe that I have an appropriate level of expertise to confirm that it is of an acceptable scientific standard.

# Reviewer report: Reviewer response for version 1

**DOI:** 10.5256/f1000research.147594.r243082

**Published:** 2024-02-20

**Authors:** Rui Santos

**Affiliations:** 1 School of Technology and Management, Polytechnic Institute of Leiria, Leiria, Portugal; 2 Integrated Researcher at the Statistics and Applications Center of the University of Lisbon, Lisboa, Portugal

The authors present a retrospective study of the etiology and the type of maxillofacial injuries depending on factors such as sex, age and monsoon season in India.

 In this study, 200 maxillofacial injuries are analyzed from the medical records of a tertiary-care dental teaching hospital. The critical analysis of the results is interesting, but there are some aspects that should be clarified and/or improved, namely:

 - The authors state "Univariate analysis was done by Student’s t-test". What was the t-test used for?

 - Tables should be presented (and numbered) in the order in which they are referred to (table 2 is referred to before table 1).

 - Figure 1 and Table 2 have the same information (and neither adds information in relation to what is in the text). I think they can be both removed.

 - All information in Table 1 is referred to in the text (this table can also be removed).

 - All information from Table 3 is included in the first lines of Table 4, so this table must be removed.

 - In Table 4, the column headings are clearer if are used in the same way as those in Table 3 (i.e., "Non-monsoon season" and "Monsoon season" instead of 0 and 1). The same occurs in the line designations, where "Absent" and "Present" are clearer than 0 and 1 (even with the explanation in a footnote to the table).

 - The results section should be developed in order to clarify the results presented, in particular which p-value is presented in Table 4 (i.e., which test was carried out, as it appears to be a comparison test of the proportions observed between the two seasons, but the only test referred in the text is the univariate t-test which is not suitable for this purpose). Moreover, in Table 4, why is "Sig" (significant) the p-value "0.053" on the "FRONTAL BONE", but the p-value "0.021" is not significant in the "ORBITAL FLOOR"?

 - Some discussion and references are related to the etiology of maxillofacial injuries, such as road traffic accidents, falls, assaults, sports injuries, and violence. Moreover, it is stated that "In this study the incidence of maxillofacial trauma due to RTA during the monsoon season was found to be lesser but the severity of trauma was found to be greater". Can the authors provide the data (for example in a table) on which they based this statement? In fact, in the Results section nothing is presented about the etiology of the maxillofacial injuries. If the analyzed data contains information about the etiology, it would be relevant to include this information in summary form in the Results section. Please note that the provided data does not contain any information about the etiology.

 - In the discussion, differences in terms of sex are also mentioned ("Men show higher incidence"...). Since the analyzed data contains information about the sex of the patients, what can be concluded from the analysed data? Is it possible to add results (in the Results section) that highlight the differences between the sexes in the maxillofacial injuries?

Is the work clearly and accurately presented and does it cite the current literature?

Yes

If applicable, is the statistical analysis and its interpretation appropriate?

Partly

Are all the source data underlying the results available to ensure full reproducibility?

Partly

Is the study design appropriate and is the work technically sound?

Partly

Are the conclusions drawn adequately supported by the results?

Partly

Are sufficient details of methods and analysis provided to allow replication by others?

Partly

Reviewer Expertise:

Statistical inference, data science, meta-analysis and epidemiology, mainly applied to the areas of Medicine and Forensic Science.

I confirm that I have read this submission and believe that I have an appropriate level of expertise to confirm that it is of an acceptable scientific standard, however I have significant reservations, as outlined above.

# Reviewer report: Reviewer response for version 1

**DOI:** 10.5256/f1000research.147594.r217295

**Published:** 2023-10-31

**Authors:** Amit Sethi

**Affiliations:** 1 Department of Oral and Maxillofacial Surgery, Henry M. Goldman School of Dental Medicine, Boston University, Boston, Massachusetts, USA

The authors have done well to highlight the influence weather and environmental conditions can play on the number and type of maxillofacial injuries seen.

 The authors have brought up a very unique factor which could influence the number of patients presenting with maxillofacial trauma. The authors have also done well to classify patients based on age, sex and type of injury. Sex of patients may be important and evolve in coming years as more women take up driving.

 The authors mention that the effect of rains may be mitigated by the fact that some people choose to stay home because of impending rain. This study is a good pilot study for a more elaborate study to be done in which a) duration can be longer b) the years with "good" or "bad" monsoon can be studied and data analyzed c) comparison can be done across different hospitals situated in places with light / moderate / heavy rain and then compare the variability in type and number of patients coming to emergency rooms with maxillofacial trauma

 Overall the article is a good read and discusses an important topic in an objective manner.

Is the work clearly and accurately presented and does it cite the current literature?

Yes

If applicable, is the statistical analysis and its interpretation appropriate?

Yes

Are all the source data underlying the results available to ensure full reproducibility?

Yes

Is the study design appropriate and is the work technically sound?

Yes

Are the conclusions drawn adequately supported by the results?

Yes

Are sufficient details of methods and analysis provided to allow replication by others?

Yes

Reviewer Expertise:

Dentoalveolar surgery, Anesthesia, Medicine, Trauma

I confirm that I have read this submission and believe that I have an appropriate level of expertise to confirm that it is of an acceptable scientific standard.
